# A Case of Autoimmune Neutropenia in a Patient Undergoing Breast Cancer Surgery

**DOI:** 10.1155/2024/5354241

**Published:** 2024-08-24

**Authors:** Mio Adachi, Goshi Oda, Masatake Hara, Yuichi Kumaki, Tomoyuki Fujioka, Toshiyuki Ishiba, Ukihide Tateishi

**Affiliations:** ^1^ Department of Breast Surgery Tokyo Medical and Dental University Hospital, Yushima 1-5-45 Bunkyo-ku, Tokyo 113-8519, Japan; ^2^ Department of Radiology Tokyo Medical and Dental University Hospital, Yushima 1-5-45 Bunkyo-ku, Tokyo 113-8519, Japan

## Abstract

Autoimmune neutropenia (AIN) is an extremely rare condition, and there is no effective treatment option for this disorder. AIN can cause major complications in patients with perioperative infection. Herein, we present a 56-year-old female patient who was scheduled for breast cancer surgery. However, she was unexpectedly diagnosed with AIN. Thus, the surgery was postponed, and endocrine therapy was started. After 7 months of treatment, the surgery was performed. Granulocyte colony-stimulating factor was administered before the surgery, but the patient's neutrophil count did not increase. Thus, levofloxacin was administered during the surgery. The patient had fever (38.6°C) 1 day after the surgery. Her surgical wound did not present with redness, and there were no other signs of infection. The fever subsided on the second day after the surgery. Nevertheless, antibiotics were administered for 5 days. The patient was discharged on the sixth day after the surgery.

## 1. Introduction

Neutropenia is a condition in which the absolute neutrophil count is <1.5 × 10^9^/L. Autoimmune neutropenia (AIN) is a disorder characterized by the presence of autoantibodies directed against neutrophils, thereby resulting in increased neutrophil destruction. Further, it is caused by the production of antineutrophil antibodies [1]. This disorder is classified into primary and secondary AIN. The former predominantly occurs in infancy. Meanwhile, the latter is common among adults and is caused by systemic autoimmune diseases (such as Felty syndrome, systemic lupus erythematosus, and rheumatoid arthritis attributed to infectious agents such as parvovirus B19), human immunodeficiency virus with hematologic malignancies such as B cell lymphoma and T cell disease, and noninflammatory diseases. Large granular lymphocytic leukemia/lymphoma with hematologic malignancies and drug-related effects is thought to be the cause [2].

AIN is extremely rare, and previous reports on surgery in patients with AIN are limited. In a case report, a 59-year-old male patient with AIN underwent gastric cancer surgery. After the procedure, the patient exhibited high-grade fever. However, he recovered from the infection [3]. There are no reported cases of breast cancer surgery in a patient with AIN.

## 2. Case Presentation

A 56-year-old previously healthy Japanese premenopausal woman underwent medical examination. Results revealed masses at the right breast and bilateral axillary lymphadenopathy in November 2018. The patient did not present with associated skin changes or nipple discharge. Moreover, she had no medical history of breast abnormalities and family history of breast or ovarian cancer.

**Figure 1:**
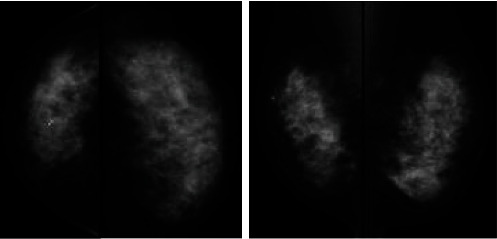
Mammography revealed distortion at the outer lower aspect of the right breast. Based on the findings, breast cancer was suspected.

**Figure 2:**
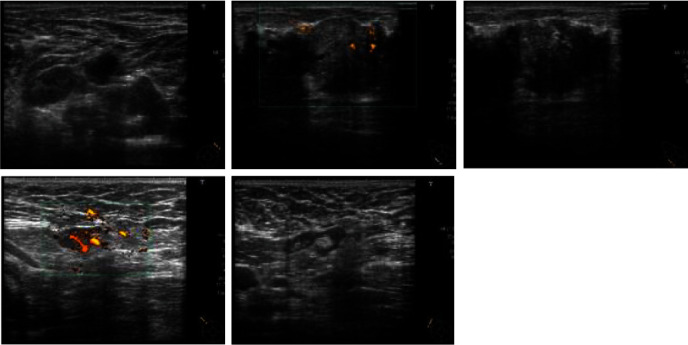
Ultrasonography revealed an irregular hypoechoic lesion at the lower outer aspect of the right breast, with a maximum diameter of ~39 mm, and bilateral lymphadenopathy.

**Figure 3:**
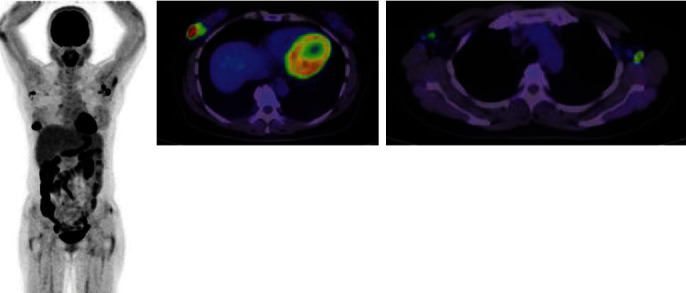
Positron emission tomography with fluorodeoxyglucose revealed fluorodeoxyglucose accumulation in the bilateral axial and pelvic lymph nodes.

**Figure 4:**
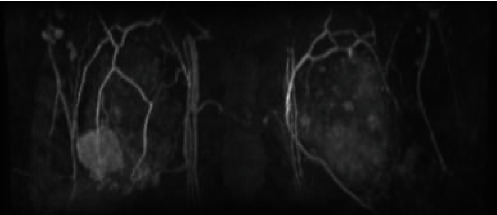
Magnetic resonance imaging revealed a fast plateau pattern with a mass measuring 35 mm in the right breast and bilateral lymphadenopathy.

Mammography revealed distortion at the outer lower aspect of the right breast (Figure 1). Ultrasonography revealed an irregular hypoechoic lesion in that area, with a maximum diameter of ~39 mm, and bilateral lymphadenopathy (Figure 2). Biopsy showed invasive ductal carcinoma with estrogen and progesterone receptor positivity, and staining yielded a human epidermal growth factor receptor 2 score of 1+. Positron emission tomography with fluorodeoxyglucose was performed and revealed fluorodeoxyglucose accumulation in the right breast and bilateral axial and pelvic lymph nodes (Figure 3). Magnetic resonance imaging revealed a fast plateau pattern with a diameter of 35 mm in the right breast and bilateral lymphadenopathy (Figure 4). The patient's white blood cell (WBC) count was 1.6 × 10^9^/L, and her absolute neutrophil count (ANC) count was 0.3 × 10^9^/L (Table 1). The enlarged lymph nodes in the axilla and pelvis were suspicious for lymphoma. Left axillary lymph node biopsy was performed, and the results did not indicate malignancy. Therefore, the diagnosis was T2N0M0 stage IIA breast cancer. The enlarged lymph nodes were attributed to susceptibility associated with neutropenia. The cause of neutropenia was unknown. Thus, breast cancer treatment with anastrozole and a luteinizing hormone-releasing hormone (LH-RH) agonist was started.

A hematologist was consulted about the unexplained neutropenia. For the time being, treatment with anastrozole and LH-RH agonists was initiated. After examining immunoglobulin genes and T cell receptor genes via Southern blotting, no gene rearrangements were detected, and malignant lymphoma was ruled out. Then, hepatitis B and C viruses and human immunodeficiency virus infections were also ruled out. The patient did not present with recent episodes of infection, thereby ruling out any infection as a cause of neutropenia. Bone marrow biopsy was performed. Results showed that the proportion of blast cells did not increase and that the karyotype level was normal. Myelodysplastic syndrome was not observed. Further, collagen diseases such as systemic lupus erythematosus and Sjögren's syndrome, which can cause neutropenia, were ruled out (Table 2). Immune-mediated neutropenia was suspected, and the patient's serum was tested for neutrophil antibodies. The test results revealed antigen HNA-1 positivity (Table 3). Hence, the patient was diagnosed with AIN, which explained the challenging recovery from neutropenia.

**Figure 5:**
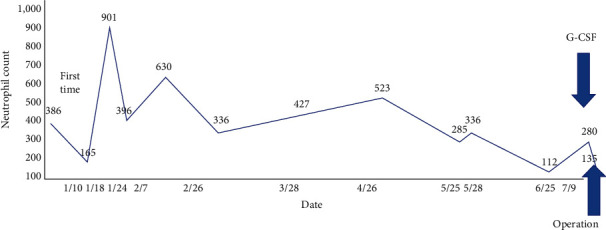
Initially, the patient's neutrophil count was 386 × 10^12^/L. It was <1,000 × 10^12^/L during the whole treatment duration. It did not increase even after the administration of granulocyte colony-stimulating factor (G-CSF).

Neutropenia was not secondary to other conditions, and further improvement in the neutrophil count was not expected. Hence, surgery was performed after 7 months of endocrine therapy. Granulocyte colony-stimulating factor (G-CSF) was planned to be administered if the patient's ANC will be <500 × 10^9^/L before surgery. Further, the patient would receive broad spectrum antibiotics during surgery in case of infection and *γ* globulin preparation if the infection could not be controlled (Figure 5).

Before surgery, magnetic resonance imaging revealed that the mass size decreased (with a diameter of 24 mm). G-CSF (150 *μ*g) was administered before surgery, and the patient's ANC decreased from 0.28 × 10^9^/L to 0.135 × 10^9^/L. Hence, G-CSF was not effective in increasing ANC.

**Figure 6:**
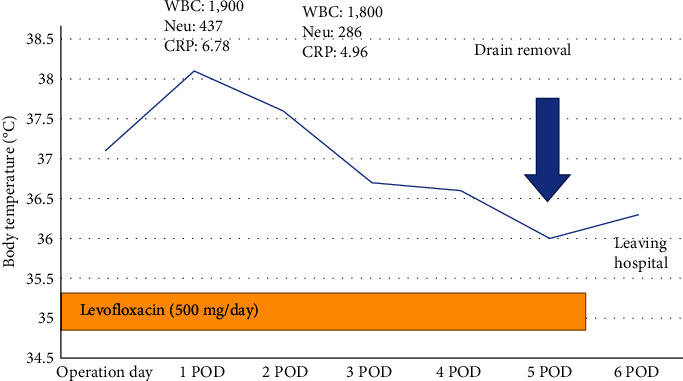
One day after the surgery, the patient had fever (38.6°C), which subsided on the next day. Five days after the surgery, the drain was removed, and antibiotic treatment was discontinued. The patient was discharged 6 days after the surgery.

Total mastectomy with sentinel lymph node biopsy was performed. Sentinel lymph node malignancy was not detected. Levofloxacin was administered during the surgery. The patient had fever (38.6°C) 1 day after the surgery. Moreover, her WBC count, ANC count, and C-related protein (CRP) level were 1.9 × 10^9^/L, 0.4 × 10^9^ /L, and 6.78 mg/dL, respectively. The surgical wound did not present with redness, and there were no other signs of infection. The fever and high CRP levels were attributed to surgical invasiveness. The patient's fever subsided 2 days after the surgery. Then, at 3 days after the surgery, the patient's WBC count, ANC count, and CRP level were 1.8 × 10^9^/L, 0.3 × 10^9^/L, and 4.96 mg/dL, respectively. The drain was removed 5 days after the surgery, and levofloxacin was discontinued. The patient was discharged 6 days after the surgery without complications (Figure 6). Antibiotics were administered for a longer period of time, and the patient was monitored for fever. No unusual management of the wound or drainage management was done during the perioperative period. The final pathological diagnosis was invasive ductal carcinoma with cancer-free margins of >1.5 cm. The patient did not present with metastases based on the sentinel lymph node biopsy. Further, she presented with estrogen receptor positivity, progesterone receptor negativity, and a human epidermal growth factor receptor 2 score of 0. The pathological stage was pT2, pN0, and pMX. The patient was premenopausal. Therefore, treatment with anastrozole and LH-RH agonist was continued after the surgery. In addition, recurrence was not observed in 3 years after the surgery. No treatment was given for AIN, and the patient was followed up.

## 3. Discussion

Herein, we report a case of AIN in a patient with newly diagnosed breast cancer. Although the patient presented with fever postoperatively, she did not develop any major infections. The diagnosis of AIN took time. Hence, breast cancer progression was suppressed with endocrine therapy. The patient's current neutropenia was diagnosed as primary AIN based on various examinations and its clinical course.

AIN is classified into primary (idiopathic) AIN, which is usually found in one per 100,000 newborns and secondary AIN, which is more common in adults. The diagnosis of this disease requires the detection of antineutrophil antibodies in the serum or neutrophil-attached antibodies against neutrophil antigens. HNA is classified into five types. HNAs 1 and 2 generally account for majority of the cases. However, in previous report, only HNAs 1 and 2 were tested [4, 5]. Secondary AIN can be associated with collagen disorders, viral infections, and lymphoproliferative neoplasms. Similar to autoimmune hemolytic anemia (AIHA) and immune thrombocytopenia, secondary AIN may precede, accompany, or follow underlying disorders. Secondary AIN is usually accompanied by AIHA or immune thrombocytopenia, and it occasionally manifests as isolated neutropenia [6, 7]. In a retrospective study of Hodgkin lymphoma with AIN, four (80%) of five patients had concurrent AIHA, Evans syndrome, or aplastic anemia [8].

One study has reported about breast cancer treated with chemotherapy. A 44-year-old woman with triple-negative breast cancer and primary AIN received neoadjuvant therapy. However, chemotherapy was discontinued due to the development of several adverse effects. Eleven months after chemotherapy, she died because of recurrent metastatic disease [9]. Mariko et al. [3] reported a 59-year-old man with gastric cancer operation and AIN. After the surgery, he developed high-grade fever. The patient recovered from infection after the readministration of G-CSF [3].

There is no fundamental treatment for AIN. However, neutropenia can only improve with G-CSF [10]. G-CSF can be extremely useful in causing temporary neutropenia remission [11]. In our case, G-CSF was administered before the surgery. However, the patient's WBC count did not increase. Thus, G-CSF was not administered during the surgery. It was unclear why neutrophil counts did not increase despite the use of G-CSF. However, since there was no other treatment option, the decision was made to continue with the surgery. Azuma et al. [12] reported the use of trimethoprim-sulfamethoxazole (TMP/SMX) during infection with AIN. TMP/SMX has a broad spectrum of activity against bacteria. None of the previous reports have addressed the efficacy of antibiotic prophylaxis in adults with AIN.

Breast cancer surgery is less invasive than other surgeries performed under general anesthesia, and the risk of major infection is usually low. However, if neutropenia of unknown cause is observed, as in this case, surgery may lead to serious infections. If such a case is encountered, based on disease biology and stage, surgery can be performed safely by consulting a specialist department.

## 4. Conclusion

To the best of our knowledge, this is the first case report of breast cancer surgery in a patient who presented with AIN. The diagnosis and treatment of AIN took time. Nevertheless, the treatment was successful, and cancer surgery was eventually performed safely.

## Figures and Tables

**Table 1:** Results of the first blood tests.

Blood count	Level	Biochemical examination of the blood	Level	Tumor marker level	Level
White blood cell count	1,600/*μ*L	Blood urea nitrogen level	11 mg/dL	BCA-255 level	39
Stab neutrophil count	11.0%	Cre protein level	0.71 mg/dL	NCC-ST-439 level	6.4
Segmented neutrophil count	13.0%	Total protein level	7.9 g/dL	Type I collagen level	6.8
Lymphocyte count	59.0%	Albumin level	3.7 g/dL	CA15-3 level	12.3
Monocyte count	16.0%	Sodium level	142 mEq/L	Carcinoembryonic antigen level	1.2
Eosinophil count	15.0%	Potassium level	3.9 mEq/L	—	—
Basophil count	1.0%	Chloride level	104 mEq/L	—	—
Hemoglobin level	8.4 mg/dL	Calcium level	9.4 mg/dL	—	—
Hematocrit count	30.1%	Lactate dehydrogenase level	177 U/L	—	—
Platelet count	26 × 10^5^/*μ*L	Aspartate aminotransferase level	19 U/L	—	—
	—	Alanine aminotransferase level	16 U/L	—	—
	—	C-reactive protein level	0.80 mg/dL	—	—
	—	Vitamin B12 level	268 pg/mL	—	—
	—	Ferritin level	22 ng/mL	—	—
	—	Soluble IL-2 receptor level	606 U/mL	—	—
	—	*β*2-microglobulin level	2.01 *μ*g/mL	—	—

**Table 2:** Test for collagen disease.

CH50 level	47 U/mL
C3 level	126 mg/dL
C4 level	20 mg/dL
Antinuclear Ab	X40
SS-A Ab	Negative
SS-B Ab	Negative
IgA level	244 mg/dL
IgG level	2,105 mg/dL
IgM level	313 mg/dL

**Table 3:** Results of the indirect immunofluorescence test.

HNA-1 type	Negative control serum (NC)	Patient (P)	P/NC	
1a/1a	2,073	7,454	3.6	Positive
1a/1a	1,232	6,281	5.1	Positive
1b/1b	821	5,990	7.3	Positive
1b/1b	2,018	4,748	2.4	Positive

## Data Availability

The datasets used and analyzed during this study are available from the corresponding author upon reasonable request.
